# The effects of low-level laser therapy on muscle strength and functional outcomes in individuals with knee osteoarthritis: a double-blinded randomized controlled trial

**DOI:** 10.1038/s41598-022-26553-9

**Published:** 2023-01-04

**Authors:** Amornthep Jankaew, Yu-Lin You, Tai-Hua Yang, Yu-Wei Chang, Cheng-Feng Lin

**Affiliations:** 1 Institute of Allied Health Sciences, College of Medicine, National Cheng Kung University, Tainan, Taiwan; 2 Department of Physical Therapy, College of Medicine, National Cheng Kung University, Tainan, Taiwan; 3 Department of Orthopedics, National Cheng Kung University Hospital, Tainan, Taiwan; 4 Deparment of Biomedical Engineering, College of Engineering, National Cheng Kung University, Tainan, Taiwan; 5 Physical Therapy Center, National Cheng Kung University Hospital, Tainan, Taiwan

**Keywords:** Health care, Medical research, Rheumatology

## Abstract

The purpose of this study was to compare the therapeutic effects of low-level laser therapy (LLLT) with 808 and 660 nm wavelength on muscle strength and functional outcomes in individuals with knee osteoarthritis (OA). A total of 47 participants were randomly assigned to the 808 nm, 660 nm, and sham control groups. Two LLLT groups received continuous LLLT with a mean power of 300 mW in different wavelengths at the knee joint 15 min a session three days per week for eight weeks, while the control group received the sham LED treatment. The knee strength and functional performance involving 30-s sit-to-stand, 40 m fast-paced walk, stair climbing, and the TUG test were measured at the baseline and one week after the interventions were completed. The results showed that knee extensor strength was more improved in the 808 nm group as compared to the 660 nm group (*p* < 0.001, *d* = 0.57) and the sham control (*p* < 0.001, *d* = 0.40), while increased flexor strength was demonstrated in the 808 nm (*p* = 0.009, *d* = 0.67) and sham control groups (*p* < 0.001, *d* = 0.97). The number of 30-s sit-to-stand was increased only in the 660 nm group (*p* = 0.006, *d* = 0.49). All three groups exhibited improvements in the other three functional performance-based tests after the interventions with no statistically significant differences among the groups. In conclusion, both intervention groups improved muscle strength and functional performance as compared to the control group. The 808 nm wavelength group showed better results in knee extensor strength. Therefore, laser therapy is suggested to be integrated into rehabilitation programs to improve muscle strength and functional performance in the population with knee OA.

## Introduction

Knee osteoarthritis (OA), a common osteopathic problem, usually occurs among people aged over 60 years old. The prevalence of knee OA is increasing substantially around the world due to the aging society^1^. Damage and loss of articular cartilage, remodeling of subarticular bone, formation of osteophytes, ligament laxity, and weakening of periarticular muscles around the knee joint are the characteristics of OA occurring as a result of disease progression. Knee OA tends to cause a significant burden to those affected, including joint pain, stiffness, and muscle weakness. These deficits often lead to limitations of movement and functional activities in the large weight-bearing joints^2,3^. Inability to voluntary and fully activate the muscles as well as a decreased muscle cross-sectional area contribute to insufficient muscle strength in the lower extremities especially the quadriceps, hamstrings, and gluteal complex^4^. Previous studies also have found that muscle weakness in the lower extremities is the main cause of functional decline and is positively correlated with the degree of disability^5,6^. Therefore, restoring the muscle strength in the lower extremities is an important factor for preventing deterioration of OA and physical activity.

The main goals of treating osteoarthritis are to control symptoms, restore joint function, and prevent progressive disease. A combination of nonpharmacological and pharmacological interventions is recommended as the first-line therapy for knee OA^7,8^. Exercise in conjunction with physical modalities such as ultrasound therapy, electrical stimulation, or laser therapy are involved in pain management and improve the level of disability in individuals with knee OA^9,10^. Low-level laser therapy (LLLT), which is non-invasive, has been shown to have significant clinical effects on pain relief through analgesic and anti-inflammatory effects. Laser therapy has been shown to have effects on restoring muscle function through the facilitation and proliferation of myosatellite cells and enhancing angiogenesis and formation of myotubes through increasing the regeneration of muscle fibers and the density of mitochondria^11^. Many techniques, including variations in energy intensity, type of radiation, and different wavelengths, are commonly used. However, the standard parameters are inconclusive and are still debated in the clinical use.

According to guidelines of the Osteoarthritis Research Society International (OARSI), a 30-s chair stand test, a 40 m fast-paced walk test, a stair climb test, and the timed up and go test are recommended as standard outcome measures among studies on this topic^12^. These examinations are used to represent the daily physical activities including ambulation, stair climbing, and sit-to-stand mobility. Also, the functional performance test plays a crucial role in treatment goals and rehabilitation progression^12^. To ensure that the measurement outcomes across different studies can be compared, in this study, the recommended functional tests were employed as one of the outcome measures for the purpose of investigating the effectiveness of LLLT among knee OA participants.

LLLT has shown to be effective in terms of pain reduction and functional improvement. However, the absorption ability of tissue differs depending on the wavelength, as confirmed in a previous study^13^, so the effects of different wavelengths of the LLLT on muscular strength and functional activities for individuals with knee OA are limited. Therefore, the purpose of the current study was to compare the therapeutic effects of LLLT on knee muscle strength and functional performance in individuals with knee OA taking into consideration different laser wavelengths. We hypothesized that the LLLT within the infrared fields (808 nm wavelength) would demonstrate better treatment effects on muscle performance leading to functional improvement than the red field (660 nm wavelength) or the sham treatment.

## Methods

### Study design

A double-blinded randomized controlled trial was designed for the purposes of this study. During the first visit, participants were screened and randomly allocated into three groups: LLLT with a wavelength of 808 nm, LLLT with a wavelength of 660 nm, and the sham control group with LED red light. The randomization was performed by the researcher (Y.L.Y.) based on the block randomization method. The block size was 3 for 1:1:1 randomization of 3 groups. Three laser machines were set with the parameters and labeled with only numbers 1 to 3 before the experiment started. Thus, both participants and the examiner were blinded to the input settings of all machines. Only the researcher who was in charge of data processing and analysis took responsibility for the setup information. In addition, the same orthopedic surgeon evaluated the K-L radiographic scale grades while the same licensed physical therapist took the responsibility for applying the laser machines and the evaluation and assessment. The study protocol was prospectively registered to ClinicalTrials.gov before the first participant was enrolled. The clinical trial number was NCT04828252 (Trial registered on 03/02/2021 and posted on 02/04/2021). Prior to the data collection, the participants were informed of the full treatment protocol and signed an informed consent approved by the Institutional Review Board (IRB) of National Cheng Kung University Hospital following the declaration of Helsinki. The ethics protocol number was A-ER-109-187.

### Participants

The sample size estimation required in the present study was computed in a G*Power 3.1.7 program. The sample size was calculated based on a pilot result, which indicated a minimal level of detectable change for the self-paced walking time test. The calculated effect size was 0.8 (medium to large effect size). Therefore, at least 46 participants were required to achieve an 80% statistical power with an alpha level of 0.05 for a dependent mean study design.

**Figure 1:**
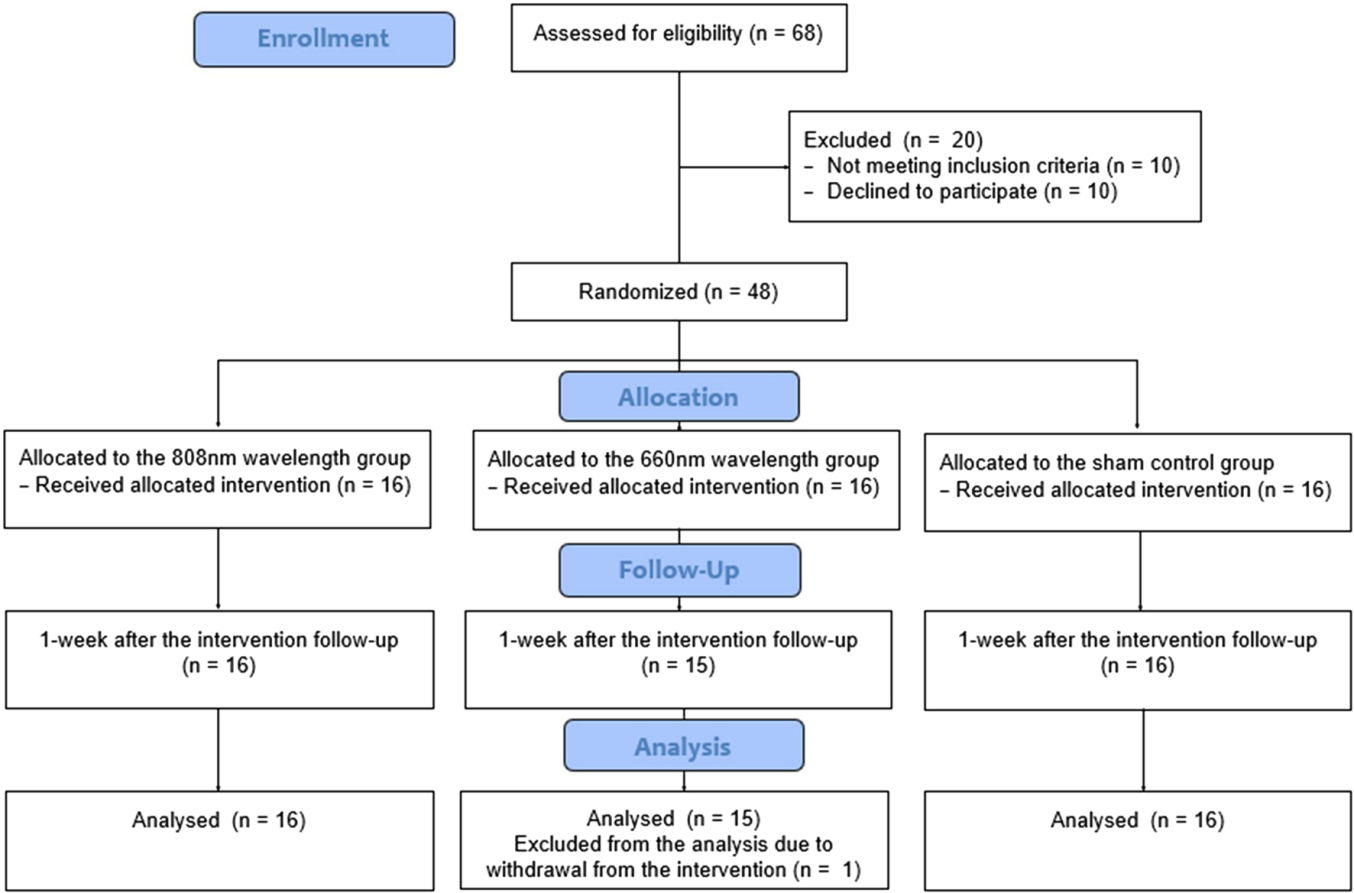
CONSORT flowchart of the study.

A total of 48 knee osteoarthritis participants were screened and recruited from the university hospital and local orthopedic clinics. The inclusion criteria included (1) knee OA patients diagnosed by orthopedic surgeons in both genders (aged between 50 and 80 years old) with K-L radiologic scale grades 2 and 3, (2) experiencing knee pain ≥ 3 on the visual analog scale (VAS, ranging from 0 to 10), (3) able to walk on level ground and able to climb stairs without an assistive device or assistant, and (4) not enrolled in intensive exercise or rehabilitation programs. The exclusion criteria included (1) acute infectious diseases, (2) abnormal blood pressure or fever, (3) tumor or cancer patients, (4) pregnant, (5) special abnormalities or paresthesia, (6) coagulation disorders, (7) implantable devices such as pacemakers or EKG machines, and (8) previous surgery in the knee or hip with total or partial prosthesis of the knees or hips. The CONSORT flowchart of the study is summarized and shown in Fig. 1.

### Procedure

Participants were assessed for lower extremity muscle strength and functional outcomes for the pre-intervention test. Then, the participants were randomized into the groups and underwent the eight-week intervention program. The post-test was carried out following by the same sequences as the pre-test. To prevent cross-over effects, all included participants were prohibited from engaging in other rehabilitation programs during the protocol period. Also, on the pre-and post-assessment day, the participants were requested to avoid alcohol and to refrain from caffeine and consumption of painkillers.

### Outcome assessments

The Myoflet3 handheld dynamometer (Boston, USA) was used to assess lower extremity muscle strength as the primary outcome in this study. The muscle strength test was started with knee flexors in the prone position with a knee flexion of 90°. The knee extensor test was performed in the short sitting position with arms across the chest to prevent compensatory movement. The strength test was performed three times in each position, and the average values were reported as the muscle strength.

Performance-based measures of function were performed as recommended by the OARSI^12^. Four performance-based tests were administered in a sequence from less to more vigorous-intensity physical activity so as to eliminate the fatigue effect. The tests were performed three times for each performance test starting with the 30-s sit-to-stand test, the 40 m fast-paced walk test, the TUG test, and the stair climb test. In the 30-s sit-to-stand test, the participants were seated on an adjusted chair with their arms crossed over the chest. Then, they were asked to stand until their hips and knees were fully extended and then to return to the initial seated position, repeating safe movement as many times as possible and as quickly as possible within a 30 s period. The total number of correct stands was recorded within 30 s. In the 40-m self-paced walk test, the participants were asked to walk along a 10-m walkway as quickly but as safely as possible from the start line to the 10-m mark and then to circle around a cone and to return to the starting line three times (a total distance of 40 m). The time was recorded when the participants start walking and was stopped when the participant’s first heel completely crossed the starting line. In the timed up and go test, the participants were asked to sit on an adjusted chair. Then, they were asked to stand up, walk as quickly as possible to a cone 3 m away, turn around, walk back, and sit in the initial seated position. The time was recorded when the participants started standing up from the chair and was stopped when the participants returned to a sitting position on the chair. In the stair climbing test, the test was administered using 13 stair steps with a 180-cm stair height. The participants were instructed to climb up the stairs, turn around, and climb down as quickly as possible while still ensuring safety. The time was recorded when the participants started ascending the first step and stopped once the participants returned to the starting point.

### Intervention

**Figure 2:**
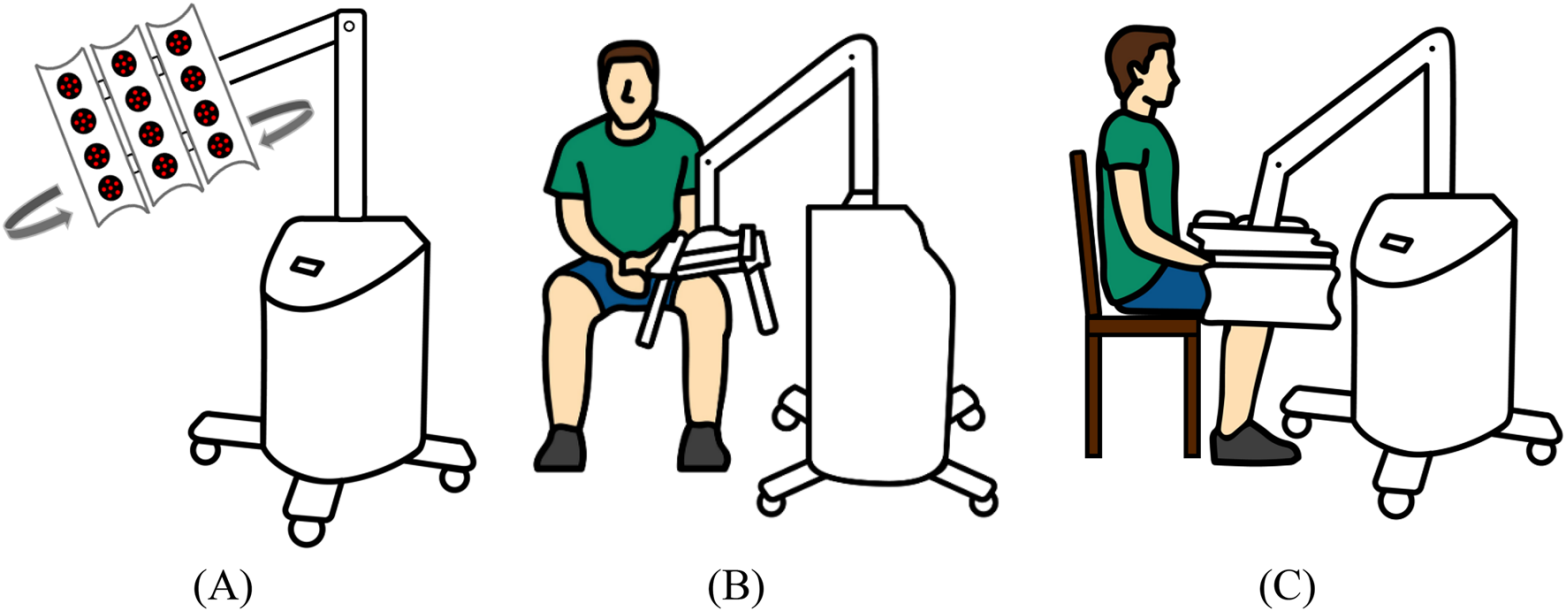
Laser machine (**A**) and participants’ position in anterior view (**B**) and lateral view (**C**).

The LLLT of each group was performed at the knee joint in a sitting position three times per week for 8 weeks with no additional exercises or other interventions. Each machine has 3 panels with 4 treatment spots/panel as inversed U shape to cover each knee joint and give the energy of 5.76 J. This dosage met the minimum dosage recommended by the World Association for Laser Therapy for knee OA (a minimum dosage of ≥ 4 J per treatment point for the 808 nm wavelength but no recommendation for the 660 nm wavelength in guideline)^14^. The synovial and joint line of the knee was covered with estimated 6 treatment spots. The laser device was applied in the continuous mode with estimated 5 cm away from the surface of the knee joint (Fig. 2). The patients in group 1 received LLLT (TI 816-8C-808, Transverse Industries Co., LTD, Taiwan) with a wavelength of 808 nm, mean power output at 300 mW, for 15 min a session. The patients in group 2 received LLLT (TI 816-8C-660, Transverse Industries Co., LTD, Taiwan) with a wavelength of 660 nm, mean power output at 300 mW, for 15 min a session. The sham control group received LED red light with mean power output at 0.35 mW and 0.0033 J for 15 min a session with very limited photobiomodulation effects. The LLLT was applied for the affected side or individually applied for each knee if participants were bilaterally affected. All participants received the post-intervention assessment within a week after completing the 8-week intervention.

### Statistical analysis

Descriptive statistics were used to present the participants’ characteristics. An independent *t* test was used to calculate the baseline characteristics among the three groups. The analysis was performed based on the per-protocol analysis. The Shapiro–Wilk test was used to test the normality of the data, and a paired *t* test or Wilcoxon signed-rank test, determined based on the result of the normality of the data with *p* > 0.05, was used to compare the differences before and after the intervention in each group. Multivariate analysis of covariance (MANCOVA) was used to compare the differences among the three groups after the intervention, and the pre-assessment data were used as covariates to correct the post-assessment results. Cohen’s *d* was computed to represent the magnitude of the effect size between group differences. The statistical analysis was performed with SPSS version 22 (New York, USA) with significance set at *p* < 0.05.

### Ethics approval and consent to participate

All recruited participants provided written informed consent in accordance with the Declaration of Helsinki. The study protocol was approved by the Institutional Review Board of National Cheng Kung University Hospital. The ethical approval number was A-ER-109-187. The study was prospectively registered to *ClinicalTrials.gov* in February 2021 (Trial Registration Number: NCT04828252).

## Results

### Characteristics of the participants

A total of 48 participants with degenerative knee osteoarthritis were enrolled in the intervention (16 participants in each group). One case from the 660 nm group withdrew from the study due to pandemic outbreak in local area. Therefore, a total of 47 participants were pooled into the statistical analysis. All of the outcome measures were compared at the baseline and the results showed no statistically significant differences between the three groups. The characteristics of the participants are presented in Table 1.

**Table 1:** Characteristics of the participants in each group.

	808 nm group (N = 16)	660 nm group (N = 15)	Sham control group (N = 16)	*p*-value
**Gender**	1 M 15FM	3 M 12FM	5 M 11FM	
**Age (years)**	67.44 (6.54)	67.68 (7.81)	71.63 (7.60)	0.181
**Height (cm)**	157.13 (5.71)	159.69 (6.36)	156.19 (6.53)	0.293
**Weight (kg)**	57.50 (9.51)	61.56 (8.00)	62.38 (8.66)	0.271
**Bilateral knee OA**	12/16	14/15	11/16	
**K-L grade**	II: 8	II: 10	II: 7	
	II + : 1	II + : 2	II + : 3	
	III: 7	III: 3	III: 6	
**Muscle strength**				
Knee extensor (%improve)	24.62 (9.09)	29.95 (10.46)	26.49 (7.85)	0.272
Knee flexor (%improve)	15.54 (5.85)	17.15 (5.75)	15.72 (4.81)	0.676
**Functional outcomes**				
30-s chair stand (time/30-s)	17.08 (4.00)	16.69 (4.30)	17.19 (5.40)	0.951
40 m fast-paced walk (sec)	30.45 (3.14)	32.45 (7.35)	31.81 (5.85)	0.600
Time up-and-go (sec)	7.06 (1.53)	7.20 (1.71)	7.25 (1.43)	0.940
A stair climb (sec)	15.38 (3.08)	16.57 (5.89)	15.23 (3.62)	0.648

### Muscle strength

**Figure 3:**
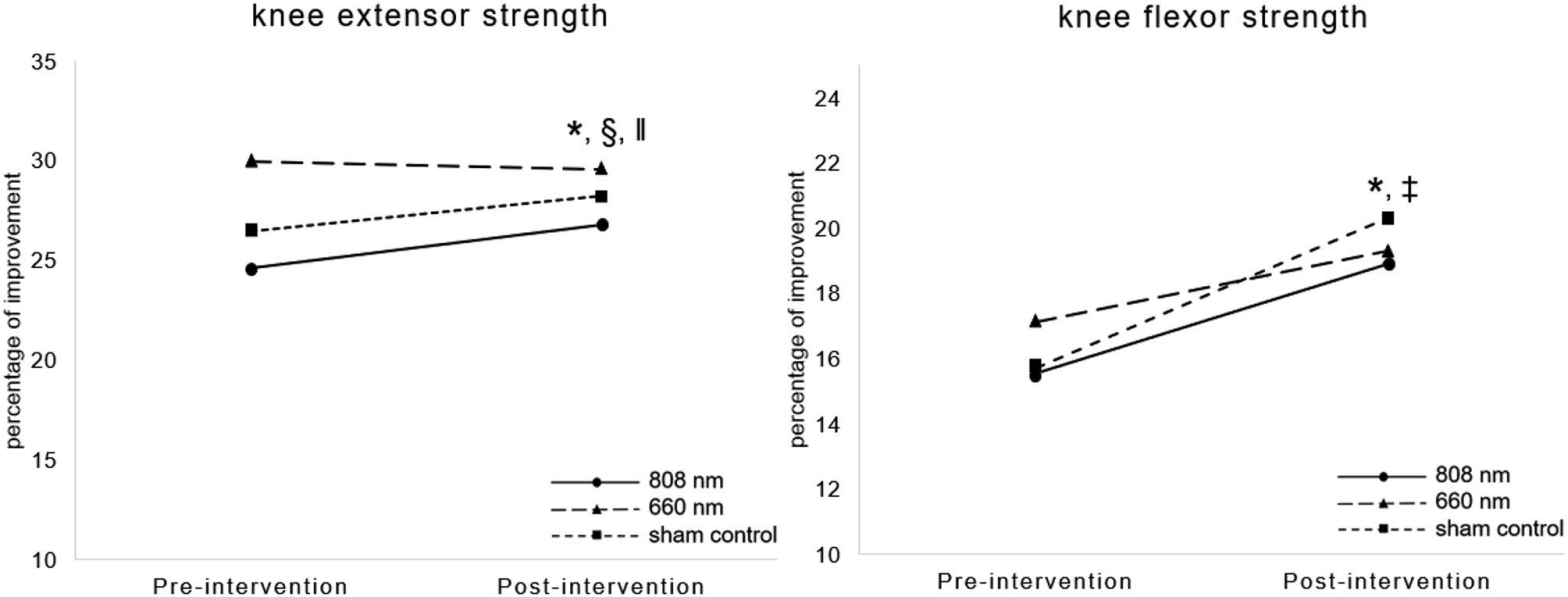
Comparison of knee muscle strength between pre-and post-intervention among the 808 nm, 660 nm, and the sham control groups. * indicates a statistically significant difference between pre-and post-intervention in the 808 nm group, ‡ indicates a statistically significant difference between the pre-and post-intervention in the sham control, § indicates a statistically significant difference between the 808 nm and 606 nm group, and ‖ indicates a statistically significant difference between the 808 nm and the sham control group.

After the 8-week intervention, improvements in the strength of the knee extensor were found in the 808 nm group only (+ 2.16%, *p* < 0.001), while the strength of the extensor showed an increasing trend in the sham control group (+ 1.74%, *p* = 0.083) and no change in the 606 nm group (− 0.36%, *p* = 0.82), as shown in Fig. 3. In addition, the ANCOVA, which was used to compare the progression of the strength of the knee extensor, revealed that the improvement in the 808 nm group was statistically significant different in terms of the group differences after the intervention (F (2, 43) = 17.988, *p* < 0.001). The post-hoc analysis revealed the differences between the 880 nm vs. the 660 nm group (*p* < 0.001) and the 880 nm vs. the sham control group (*p* < 0.001). In terms of the knee flexor strength, significant differences in the values before and after the intervention were found for the 808 nm group (*p* = 0.009) and the sham control group (*p* < 0.001) with no statistically significantly among-group differences in the ANCOVA (F (2, 43) = 1.209, *p* = 0.308, Fig. 3).

### Functional outcomes

**Figure 4:**
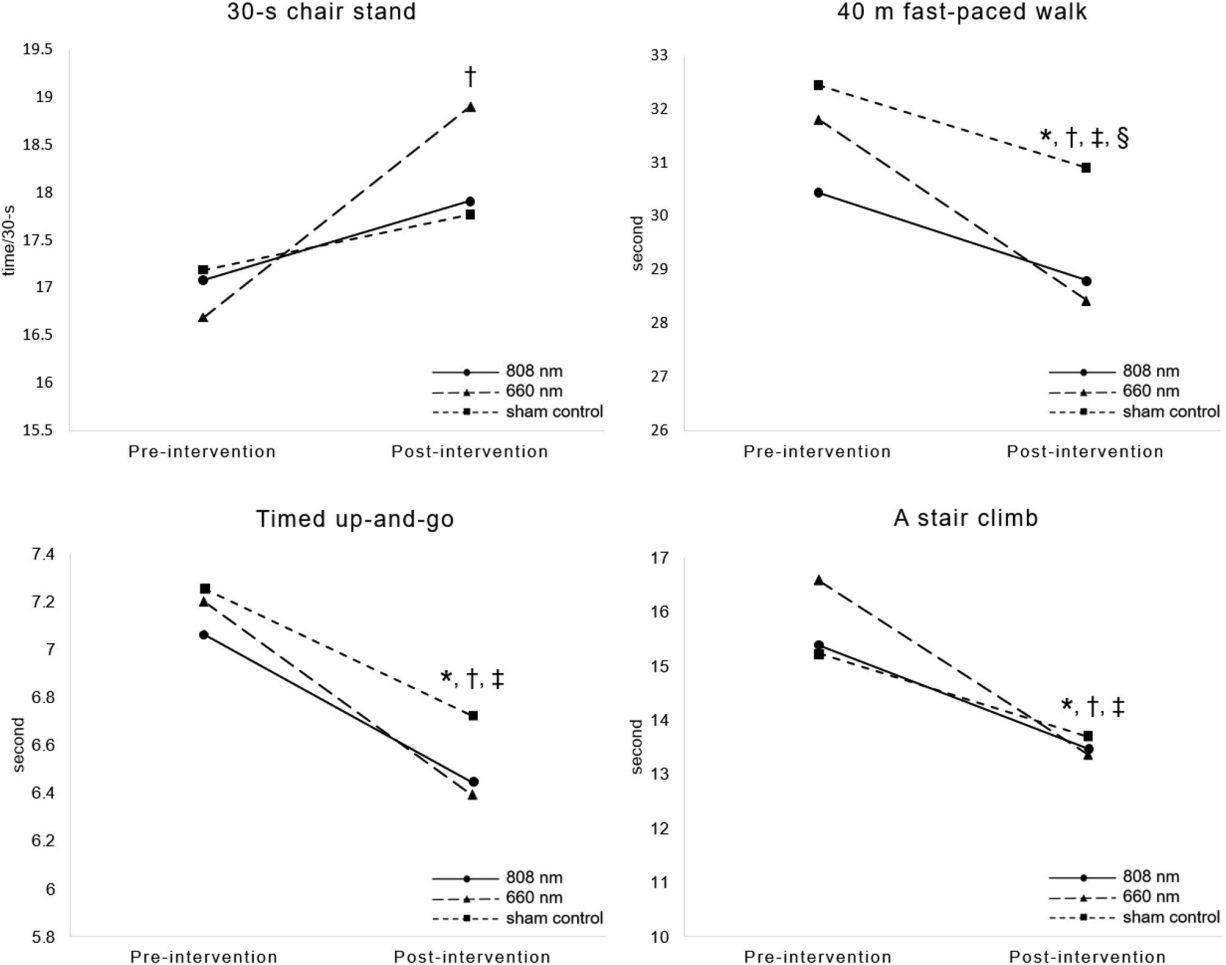
Comparison of the pre-and post-intervention functional performance-based test for the 808 nm, 660 nm, and the sham control groups. * indicates a statistically significant difference between pre-and post-intervention for the 808 nm group; † indicates a statistically significant difference between the pre-and post-intervention for the 660 nm group; ‡ indicates a statistically significant difference between the pre-and post-intervention of the sham control, and § indicates a statistically significant difference between the 660 nm and the sham control group.

An increase in the scores on the 30-s chair stand test were observed only in the 660 nm group (+ 2.22 times, *p* = 0.006), while the other two groups showed a minimal improvement (+ 0.83 times and *p* = 0.35 in the 808 nm group and + 0.58 times and *p* = 0.325 in the sham control group). Decreases in 40 m fast-paced walking time, TUG time, and stair climbing time were significantly after the intervention in the 808 nm group (*p* = 0.001, 0.009, 0.003), the 660 nm group (*p* = 0.001, 0.001, 0.003), and the sham control group (*p* = 0.009, 0.006, 0.001), respectively. Additionally, the ANCOVA statistical analysis indicated that the results for the 40 m fast-paced walking test was only significantly different among the groups after the intervention (F (2, 43) = 3.273, *p* = 0.048). The difference between the 660 nm group and the sham control group at *p* = 0.015 was investigated. A summary of the functional performance-based tests between pre-and post-intervention among the three groups is shown in Fig. 4.

## Discussion

Laser therapy has been broadly used as a physical modality in clinical rehabilitation^10^. Laser treatment provides various physiological effects for pain management and supports functional activities in knee OA patients^15,16^. This is the first study that measures the LLLT in different wavelengths on muscle strength and functional activity among mild to moderate knee OA patients. The findings of this study support the hypothesis positing that LLLT at different wavelengths aids knee muscle strength and functional performance when tested as recommended by the OARSI for individuals with knee OA^12^. The group treated at the 808 nm wavelength showed superior treatments effects as compared to the other two groups after the 8-week intervention. Additionally, no adverse events were observed or reported in this study.

Reductions in knee strength and cross-sectional areas have been well documented in OA patients. The weakness of the knee flexor and extensor contributes to poor dynamic stability and knee control^17^. This study proved that applying isolated LLLT at the knee joint can restore knee extensor and flexor strength. Physiological effects of the laser were provided among the evidence. Laser radiation can directly project to muscle cells and significantly increase the ATP synthesis activities of mitochondria and can stimulate cytochrome oxidase enhancements to mitochondria activity and aerobic capacity, in turn facilitating power generation in muscle fibers^18,19^. The findings from this study are in agreement with previous studies where a focal LLLT was applied directly to either the joint line^20,21^ or the quadriceps muscles^22^.

The results showed that the use of the 808 nm wavelength led to superior beneficial effects in terms of increasing knee extensor strength as compared to the 660 nm wavelength and the sham control. Also, the 660 nm wavelength did not show therapeutic effects on muscle strength. The differences in physiological and therapeutic effects can be explained in terms of the relationship between photon impingement and biological tissues. When photons hit biological tissue, their onward transportation depends on the reflectance, scattering, and absorption effects of the materials. Hence, various wavelengths lead to different absorption abilities. The spectrum of the 808 nm wavelength was in the infrared fields and the 660 nm wavelength is in the red fields. It is well established that cytochrome C oxidase, the terminal enzyme of the respiratory chain in mitochondria, and proteins in the cytoplasmic membrane both absorb light in the red and infrared sections of the electromagnetic spectrum respectively. They produce similar biological effects but lead to distinct metabolic activity^13^. In addition, a wavelength within the near-infrared spectrum (800 ~ 2500 nm) can better facilitate the proliferation of osteoblasts and the decomposition of collagen, which is related to the formation of cartilage^23^. Thus, a laser with wavelengths falling in the near-infrared spectrum helps to decrease pain and improve strength better than the red fields in individuals with knee OA, as supported in the previous study, where an 810 nm wavelength was used to increase quadriceps activation^11^.

Knee pain causes difficulty in daily functional activities for knee OA patients. The adaptive mechanism during walking with an OA knee is characterized by decreased stride length, increased walking base of support, and increased time required for the double support phase in order to avoid pain provocation^24^. The results of this study pointed out that an isolated laser modality treatment had some obvious effects on walking performance. The walking speed of all groups after the intervention was higher than the reference values reported by healthy older adults aged 50–59 years old and older than 60 years old^25^. It is possible that the decreased walking time during the fast-paced walk test was attributed to decreased pain and increased knee muscle strength as a result of increased walking speed in both intervention groups. However, the laser with an 808 nm wavelength did not show superior effects to the 660 wavelengths. Improved gait parameters were also reported in a study by de Matos Brunelli Braghin et al.^20^, who used LLLT at 808 nm with 5.6 J. The authors found that eight weeks of LLLT alone can increase gait speed while LLLT plus an exercise program can better improve all gait variables, such as the cadence and duration of single affected limb support.

Sit-to-stand, stair climbing, and TUG tests require greater knee extensor strength, knee joint range of motion, and joint moment than walking. The LLLT intervention groups showed an increased score for the 30-s sit-to-stand and decreased the time required for stair climbing and the TUG. The testing time among the three groups presented the same normative scores for the 30-s chair stand test^26^ and the TUG test^27^ in community-dwelling older people. The performance time required for the stair climb test varied between studies due to the different number of stair steps and the stair height used for testing^12^. Laser treatment led to pain reduction and increases in the quadricep and hamstring co-contraction mechanism, which helps prevent excessive loading in the joint during vigorous-intensity physical activity. Hence, the participants in both intervention groups were able to perform better on the sit- to-stand test, reductions in the time required for stair climbing and TUG, which are considered to be tasks that require higher loading into the knee joint. However, a significant difference among the groups was not detected.

Interestingly, the sham control group revealed the placebo responses in the functional test through a reduction in walking time, the TUG test, and stair climbing. We assumed that there was significance in the genuine biopsychosocial phenomena when the patients received the placebo effects from the laser treatment. Clinical placebo effects can be explained in terms of release of neurotransmitters and activation in many areas of the brain, which are complex neurobiological mechanisms in the connection between the brain and the body^28^. Symptom improvement is attributable in such cases to environmental cues such as medical interventions and interaction with other patients or therapists^29^. Tétreault et al. clearly demonstrated improvements in the middle frontal gyrus brain region, as measured by functional magmatic resonance imaging, among knee OA patients who were enrolled under clinical placebo conditions for a pain-relief pill^30^. Many studies of physical agents used for knee OA, such as ultrasound treatments^31^ or electrical stimulation^32^, using a sham control as the study design also reported improvements in pain and/or function after interventions. Hence, the results of this study illustrated placebo effects of laser therapy in the sham control group.

Some limitations should be addressed in this study. Firstly, as the previous study mentioned the required number of each intervention group to detect the significant therapeutic effects (N = 30)^33^, we found that the number of the enrolled participants in this study was lower than the minimum requirement. Secondly, an isolated laser modality was implemented as the treatment in this study. Based on the literature, combining laser and other treatments such as acupuncture^34^ or exercise protocols^20,21^ may lead to better improvements in knee function. Thirdly, the study examined the outcomes only before and after the intervention. A long-term follow-up is suggested to track the residual laser effects. Fourthly, the findings of this study are based on those who were diagnosed on the K-L grades 2–3 of knee OA. These results may not be generalized to others with milder severity. Early intervention in patients with mild knee pain may lead to anecdotal responses, but early intervention can prevent degeneration and functional declines. Finally, a biomechanical analysis of the functional outcomes should be explored in a future study so as to clearly describe the treatment effects on functional performance and movement control.

## Conclusion

In this study, LLLT at different wavelengths and a sham control were examined in individuals with knee OA. The findings of the current study demonstrated that LLLT with 808 and 660 nm wavelengths is able to improve knee muscle strength and functional performance after the eight-week intervention. The group treated with the 808 nm wavelength exhibited superior physiological effects than the other two groups on muscle strength. Interestingly, improvements were also observed in the sham control group for knee flexor strength and functional performance outcomes. Therefore, LLLT is recommended for use as a physical agent concomitantly with rehabilitation programs for the management of knee OA.

## Data Availability

The datasets used and/or analysed during the current study are available from the corresponding author on reasonable request.
